# Modeling Central Nervous System Injury In Vitro: Current Status and Promising Future Strategies

**DOI:** 10.3390/biomedicines11010094

**Published:** 2022-12-29

**Authors:** Kristina Pilipović, Anja Harej Hrkać, Natalia Kučić, Jasenka Mršić-Pelčić

**Affiliations:** 1 Department of Basic and Clinical Pharmacology and Toxicology, Faculty of Medicine, University of Rijeka, 51000 Rijeka, Croatia; 2 Department of Physiology and Immunology, Faculty of Medicine, University of Rijeka, 51000 Rijeka, Croatia

**Keywords:** brain injuries, traumatic, brain ischemia/hypoxia, cell culture techniques, induced pluripotent stem cells, the central nervous system

## Abstract

The central nervous system (CNS) injury, which occurs because of mechanical trauma or ischemia/hypoxia, is one of the main causes of mortality and morbidity in the modern society. Until know, despite the fact that numerous preclinical and clinical studies have been undertaken, no significant neuroprotective strategies have been discovered that could be used in the brain trauma or ischemia treatment. Although there are many potential explanations for the failure of those studies, it is clear that there are questions regarding the use of experimental models, both in vivo and in vitro, when studying CNS injury and searching new therapeutics. Due to some ethical issues with the use of live animals in biomedical research, implementation of experimental strategies that prioritize the use of cells and tissues in the in vitro environment has been encouraged. In this review, we examined some of the most commonly used in vitro models and the most frequently utilized cellular platforms in the research of traumatic brain injury and cerebral ischemia. We also proposed some future strategies that could improve the usefulness of these studies for better bench-to-bedside translational outcomes.

## 1. Introduction

Traumatic brain injury (TBI) and cerebral ischemia are major problems causing high mortality and morbidity worldwide, and great efforts are being made to develop treatments for the central nervous system (CNS) injury-related pathologies [1].

TBI is the third leading cause of death worldwide and is a major public health problem, as it also can lead to lifelong disabilities. Estimates suggest that approximately 69 million people are exposed to brain trauma each year. Presently, there is no effective therapy that can promote brain repair or reduce post-traumatic brain damage. It is certain that a better understanding of the cellular mechanisms involved in brain damage will help in the search for neuroprotective solutions. Today, there are many experimental models of brain trauma, but the problem is that the results of studies performed with these models have not been adequately translated into human clinical trials. There is a need to develop new experimental systems that can recapitulate key processes by which the mechanical energy caused by trauma is transferred to brain cells. The second important need is to involve human brain cells to ensure better efficacy of therapeutic approaches in humans [2].

**Figure 1:**
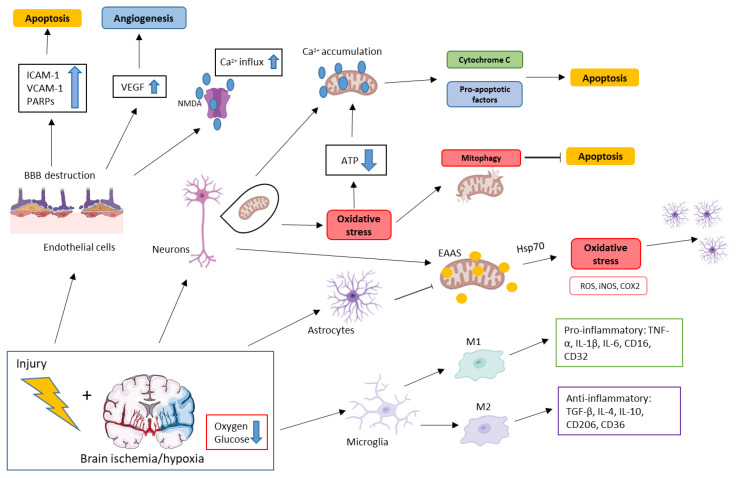
Signaling events occurring in brain cells affected by trauma and brain ischemia/hypoxia. ATP, adenosine triphosphate; BBB, blood−brain barrier; COX2, cyclooxygenase-2; EAAS, excitotoxic aminoacids; Hsp70, heat shock protein 70; ICAM-1, intercellular cell adhesion molecule-1; IL-1β, interleukin-1β; IL-4, interleukin 4; IL-6, interleukin 6; IL-10, interleukin 10; iNOS, inducible nitric oxide synthase; PARPs, poly(ADP-ribose) polymerases; ROS, reactive oxygen species; TGF-β, tumor necrosis factor-β; TNF-α, tumor necrosis factor-α; VCAM-1, vascular cell adhesion molecule 1; VEGF, vascular endothelial growth factor.

Generally, in brain trauma, injury can be divided into primary injury, which occurs after the mechanical insult, and secondary injury, which is characterized by tissue damage that ensues in minutes, hours, or even days after the primary insult [3]. Secondary injury is characterized by specific molecular pathologies in the microenvironment. These include oxidative stress, perilesional tissue architecture disturbances, and specific interactions between cells (Figure 1) [4].

Cerebral ischemia, often referred to as a stroke, is the leading cause of mortality worldwide. Annually, more than 15 million people are affected by cerebral ischemia, and 16% of humans in total have a stroke during their lifetime. The vast majority of ischemic strokes are caused by transient or permanent occlusion of cerebral blood vessels, eventually leading to brain infarction. In about 15% of cases, stroke occurs after the vessel rupture with associated hemorrhage. The main factors, on which the brain injury after ischemia depends, are the severity and duration of ischemia and the presence of the collateral blood flow [5]. In this condition, there is little time for therapeutic intervention to restore blood flow and prevent permanent brain tissue damage.

Because ischemic stroke is a rather complex condition, experimental models can only cover part of this heterogeneous disease. For the basic understanding of the main mechanisms and the major molecular pathways, in vitro studies have so far proved useful [5], but they cannot mimic the complexity of clinical stroke. Therefore, it is important to have clinically relevant models to fully understand the pathophysiology of ischemic stroke and to develop new therapeutic methods and drugs for stroke treatment [6].

## 2. In Vitro Models of Trauma

In preclinical drug development, the gold standard is still to utilize animal models, usually mice or rats, and this is also true in brain trauma research. The development of in vitro injury models for neurological diseases, more specifically for CNS trauma, usually mimics human conditions to better understand the specific elements of the injury and to test the efficacy of potentially promising medicines [4,7]. Current in vitro models for studying traumatic brain injury are not effective enough, because they do not accurately simulate all the multifaceted and heterogeneous aspects of trauma. However, in vitro models have certain advantages over animal testing, such as decreased costs, higher throughput capabilities, and in the end bigger control over experimental conditions.

Mechanical types of injuries used to mimic in vitro trauma include mechanical stretch, transection or scratch, blunt impact, and compression. The most important events included in TBI pathophysiology examined by in vitro models are membrane disruptions that lead to ionic dysregulation, then inflammation, damage of microtubules and axons, and in the end cell death. In in vitro studies, it is also important to apply controlled and repeatable injuries to cells, to adequately mimic the microenvironment of brain cells and disruptions of the mentioned microenvironment [4].

Stretch-induced injury models are the most widely used in vitro TBI models [8]. One of the simplest and accepted methods is the use of stretch in cultured murine brain cells via a cell-injury controller. Using stretch to culture brain cells helps study cellular and molecular events involved in TBI, including the blood−brain barrier (BBB) disruption. The principle of the assay is to induce injury to cultured cells through the delivery of a controlled pulse of compressed nitrogen gas to the cultured cells in the medium. Cells are cultured on specific plates with elastic membrane bottoms, and the controller enables the regulation of pressure strength, which determines the extent of injury to adhered cultured cells.

Transection and scratch injury models are used to analyze trauma-induced axotomy and to test the therapeutics efficacy of therapies aimed at promoting axonal regeneration. Primary axotomy, relatively rare in TBI in comparison to spinal cord injury, is investigated by the induction of transection injury, which not only mimics primary physical injury, but it also leads to the activation of secondary injury responses similar to in vivo conditions. The microenvironment of the injured brain is affected through the promotion of glutamate-induced excitotoxicity, release of pro-inflammatory cytokines, expression of growth factors, axonal growth-inhibiting molecules, changes in cell metabolism, and production of reactive oxygen species [9]. The scratch assay is a simple transection in vitro model where primary neurons, astrocyte cultures, or immortalized cells are scraped using a pipette tip. It is commonly used to induce astrocytic reactivity and assess the response of these cells, as they are extremely important in wound closure and healing processes [10].

One of the mechanisms involved in brain trauma pathophysiology is the process of cavitation. It is a process of vaporization, bubble generation, and bubble implosion that results from decreases and increases in pressure. The so-called “flyer-plate model” is a model that represents such a type of trauma. It is an in vitro high-energy model of trauma, and the principle of the method is to hit the bottom of a cell culture causing cavitation and consequently creating shock waves inside the well and the medium. This model is useful for analyzing the cellular responses to micro cavitation, particularly in neuronal cells [11].

A model that is also used to investigate neurotrauma is “brain-on-a-chip”. It consists of 3D cell cultures, and it is used in an effort to model the physiological responses of brain tissue in a microfluidic environment. 3D culturing methods represent a good model to determine the characteristics of the glial scar, the main feature of secondary injury. The main use of the model is the high throughput screening of compounds in pharmacological and toxicology studies. It is also a good model to study diseases process, by adding free radicals, causing inflammation or using modified cell lines to stimulate diseases. This type of model requires mechanical injury at the microscopic level of axons and neurons, so the credibility of such an injury is questionable and quite difficult to carry out [3].

Overall, in vitro CNS trauma models are very valuable in clarifying brain pathologies after the primary and secondary injury to explore new clinical treatments. Nevertheless, there are some important limitations, such as the differences between cultured cells and tissues and their matching in vivo counterparts due to variances in the microenvironment. The problem which occurs is how to prepare ex vivo brain or spinal cord slices, without damaging cells and tissues and affecting cellular and molecular responses due to experimental injury procedures and therapy treatments [12].

The other problem is how to imitate in vitro microenvironment conditions as faithfully as possible to represent in vivo state, because it is hard to predict how the patient’s cells will respond in the clinical environment. Moreover, it is difficult to determine the drug dosage for in vivo application based on in vitro drug testing. While animal trauma models will remain necessary during pre-clinical drug testing, in vitro models could be remarkably improved for different drug discovery testing. Finally, the replacement of in vivo experiments by appropriate in vitro studies would contribute to the reduction in the number of tested animals [3].

## 3. In Vitro Models of Cerebral Hypoxia/Ischemia

A cascade of cellular events that begin with a loss of oxygen, followed by energy depletion, excitotoxicity, and subsequent complex changes in tissue metabolic activity, are main characteristics of the cerebral hypoxia/ischemia pathophysiology. Intense post-ischemic inflammation is mediated by the activation of various pro-inflammatory cytokines and chemokines, and perturbed mitochondrial function is responsible for the apoptotic pathways activation [13]. Several in vivo models have been established to mimic clinical conditions of global (e.g., cardiac arrest) or focal (e.g., stroke) cerebral hypoxia/ischemia. Although in vivo models most realistically mimic the clinical conditions, including reperfusion, in vitro models of cerebral hypoxia/ischemia are important for understanding and elucidating the complexity of the pathophysiological cascade of biochemical and molecular mechanisms involved in these processes [14]. There are several possibilities to induce hypoxia/ischemia in vitro, but the most commonly used models are inhibition of cellular metabolism by chemical or enzymatic blockade and oxygen-glucose deprivation (OGD) [15]. The introduction of new technologies allows better modeling of ischemia-reperfusion injury in vitro by using OGD media flow perfusion methods or different cell culture platforms [7,16,17].

Inhibition of cellular metabolism can be triggered by various chemicals that interfere with the electron transport chain and lead to the energy deficiency that occurs in the initial phase of cerebral hypoxia/ischemia. The most used chemical inhibitors include antimycin, rotenone, 2-deoxyglucose, or sodium azide. In addition, it is possible to induce cell injury by using NMDA or glutamate receptor agonists to mimic in vivo excitotoxic conditions that result in a substantial extracellular increase in glutamate [18,19]. The enzymatic methods used to induce in vitro hypoxia/ischemia conditions are the glucose oxidase/catalase systems, consisting of catalase and 2-deoxyglucose. The advantages of both methods are their relatively simple and accessible methodology and the ability to rapidly gain insight into the specific mechanisms involved in the pathophysiological cascade during the hypoxia/ischemia process. However, in vitro assays cannot provide insight into the complexity of the processes that occur under in vivo conditions. A particular problem is the lack of appropriate possibilities to test the processes occurring during reperfusion in vivo.

**Figure 2:**
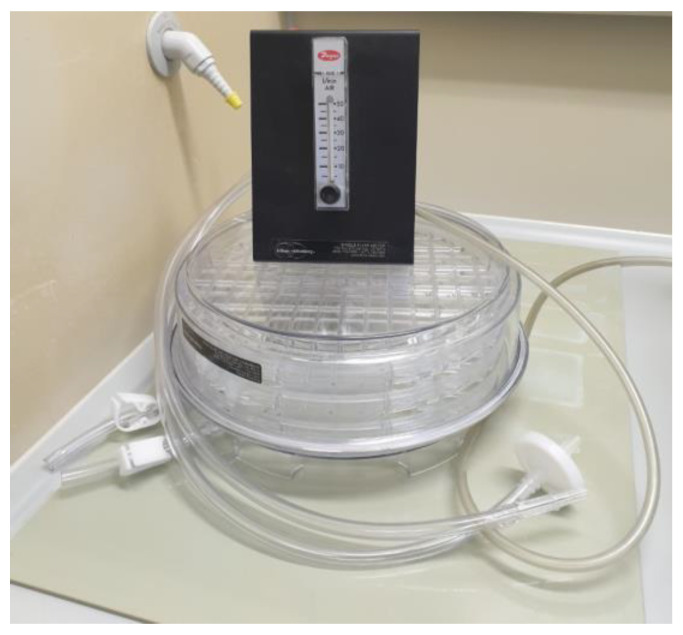
Hypoxia incubator chamber.

Oxygen-glucose deprivation (OGD) is the most commonly used and relevant method to create in vitro hypoxia/ischemia-like conditions that mimic stroke. This is usually conducted by exposing cells to glucose-free media and displacing oxygen with a nitrogen/carbon dioxide mixture in a hypoxia chamber (Figure 2). This model allows mimicking reperfusion conditions by reintroducing glucose with a return to atmospheric oxygen. OGD was described to induce neuronal depolarization within 10 min of onset. Within 30 min, there was depolarization of astrocytes and acute cell swelling followed by apoptotic and excitotoxic necrotic cell death, consistent with observations of ischemia-reperfusion injury in vivo. OGD is also associated with a sharp increase in extracellular glutamate concentration, consistent with excitotoxic effects in vivo [7,19]. Most in vitro ischemia models mimic global ischemia, because they induce an insult over an entire brain slice or plate of cultured neurons and therefore do not mimic the clinical situation of a focal insult. Another method of targeted OGD media flow perfusion was also developed in which OGD medium is focally applied to a small portion of a brain slice while the rest of the slice is bathed with a normal oxygenated medium [17]. In this model, rapid neuronal depolarization occurs in the core of the OGD target area with slower progressive depolarization in the surrounding perfused area, as seen in the ischemic penumbra.

Variable durations of OGD can been used, depending on the purpose of the studies, and OGD could be applied intermittently or continuously. Regarding the duration of OGD, long-term protocols (from 40 min up to 72 h) are used to replicate hypoxia/ischemia-like conditions. In these protocols, ischemia/hypoxia can be followed by reperfusion, which has a significant impact on the outcomes of the experiment. This is perhaps most relevant and related to the effects of reperfusion on the changes in the intracellular Ca^2+^ levels [20]. Namely, it was found that during prolonged ischemia without reperfusion, there are two phases in the [Ca^2+^]_i_ changes: hyperexcitation phase followed by the phase of global [Ca^2+^]_i_ increase. If such a longer ischemic period is followed by reperfusion, this causes an additional sharp increase in [Ca^2+^]_i_ and, subsequently, additional cell death. OGD can be used to study another experimental paradigm—the effects of and the possible neuroprotection provided by the hypoxic preconditioning. For example, these brief (3 to 10 min) hypoxic episodes have shown to alter glutamate receptor mediated [Ca^2+^]_i_ response in hippocampal neurons [21].

**Figure 3:**
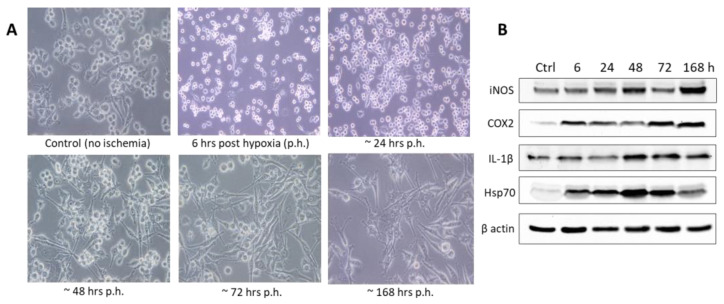
BV-2 cells under normoxic and hypoxic conditions. (**A**) Different and distinct cell morphology phenotypes of BV-2 microglia cells (from amoeboid to branched/ramified) clearly visible with the bright field /phase-contrast under normoxic and hypoxic conditions. Immediately after a 6-h-induced hypoxic condition, the amoeboid-shaped cells were predominantly present in the cell culture during the next 48 h, exhibiting changes after 72 h with the appearance of an increased number of branched cells during the 7-day culture (up to 168 h after hypoxia). (**B**) Western blot analysis of selected inflammatory and oxidative stress markers expression under normoxic and hypoxic conditions.

Experiments performed in vitro with hypoxia alone better represent cerebral hypoxic conditions such as carbon monoxide poisoning than ischemic stroke, because they mimic conditions in which blood flow is maintained [22]. Many in vitro studies have shown that hypoxia alone causes dramatic changes in endothelial cell actin cytoskeleton (EC) and tight junction protein localization in BBB models. The majority of experiments were performed with the immortalized BV-2 microglia cell line, a proven replacement for primary microglia [23]. These cells spontaneously show a dual phenotype with predominant growth in an amoeboid cell form cultured under standard conditions of 10% DMEM (DMEM supplemented with 10% *v*/*v* FCS, fetal calf serum) (Figure 3). In the presented in vitro model, hypoxia was induced in a chamber where oxygen levels were reduced to < 2% by gradually introducing 98% nitrogen and then maintained for 6 h. Microscopic analysis of BV-2 microglial cells was performed immediately and at 24, 48, 72, and 168 h after hypoxia. The responses of the cells, i.e., changes in morphological characteristics, were observed in the early time periods of the experiment, which served as indirect indicators of cell activation under hypoxia conditions. The later intervals were accompanied by a change in cell morphology, with a ramified cell form predominance as a sign of recovery from hypoxia (unpublished data).

In addition, the expression levels of oxidative stress protein markers (iNOS and COX2) as well as pro-inflammatory (IL-1β) and chaperone (hsp70) proteins were analyzed by Western blot, elucidating the hypoxia-induced changes associated with the microglia cell activation. Thus, the increased expression of these markers (Figure 3B) convincingly shows the degree of oxidative stress caused by hypoxic damage, indicating also the potential, extent, and dynamics of cellular proteomics associated with inflammatory and energy events leading to oligemic brain in ischemia/hypoxia.

## 4. Cell Culture Platforms Used in Traumatic Brain Injury and Brain Ischemia In Vitro Models

Cellular in vitro platforms used up until now to model TBI and stroke are brain slices, organotypic cell cultures, primary neuronal cells, immortalized cell lines, and different types of stem cells of human and rodent origin [2,24,25]. In the in vitro studies of TBI, most used cells are the ones of rodent origin, and of those rats account for about 70 % [2]. When it comes to the use of cells of human origin, it has been reported that they were used in only about 15% of studies. In the studies that used human cells, the researchers have most frequently used immortalized cell lines, followed by primary cells, and induced pluripotent stem cells (iPSCs).

For the in vitro stroke research, the main cellular platform is the one in which monocultures of rodent primary neurons are used, followed by organotypic brain slice cultures, while the use of co-cultures and 3D cultures as well as the cells of human origin have not been as prevalent thus far [2].

In the following subsections, we will provide an overview of the most commonly used cellular platforms in the TBI and stroke research.

### 4.1. Primary Cell Lines

Even though the use of the so-called monocultures (cell cultures that consist of a single cell type) does not provide us with the information on the complex tissue and organ reactions to noxious events or the effects of pharmacological interventions, they still give us an important insight into cell-specific responses as well as the reaction of particular cell types to neuroprotective agents. Most commonly, rodent (rats more often than mice)-derived neuronal cells are used. However, an important part of the tissue response to ischemia or mechanical damage belongs to glial cells, i.e., astrocytes, microglia, and oligodendrocytes. In studies related to the BBB reaction, endothelial cells have also been used.

From the technical standpoint, primary cell isolation and preparation can be viewed as time-consuming, and the purity of cell cultures might be challenging to achieve, and this needs to be considered regarding the reproducibility of the results. Additionally, primary cells are dissociated from either embryonic tissue or from the animals sacrificed in early postnatal days, so cells need to be maturated during a period of time.

The main advantage of using monocultures is that this method allows for a high-throughput analysis of cell specific responses to biological factors. However, it is still a poor representation of physiological responses to, for example, injury or ischemia in an in vivo state. This is mainly because of limited cell-to-cell interactions as well as because, in these conditions, there is no extracellular matrix (ECM) or cell−scaffold interfaces.

However, in the recent years, there is an increase in the number of studies in which 2D cultures were created by using human iPSCs and embryonic stem cells (ESCs), which has enhanced the scientific value of research results [26].

### 4.2. Immortalized Cell Lines

The use of established cell lines has plenty of advantages in in vitro studies. These cells are highly proliferative, and they offer high reproducibility with the possibility of easy genetic manipulation. Additionally, many of used immortalized cell lines are of human origin, i.e., with human genetic backgrounds. However, these types of cells might require differentiation protocols for them to reach necessary morphological and/or physiological characteristics. In addition, immortalized cell lines have an oncogenic origin, and their main characteristic is the high proliferation rate, something that is clearly not a feature typical for cells of the CNS origin.

As is the case with dissociated primary cultures, immortalized cell line cultures lack in that they are unable to imitate higher-dimension interactions between cells as well as to consider the influence of the ECM environment the cell reactions to injurious events. Most commonly used immortalized cell lines in TBI and stroke research are presented in Table 1.

### 4.3. Co-Cultures, 3D Culture Models, and Brain Organoids

As stated earlier, monocultures have so far been the most used for establishing in vitro platforms in TBI and stroke research. However, a step closer to creating a more physiologically complex environment, and more similar to human brain, is using multicellular and multidimensional cell culture models.

By combining different cell types, studies are able to imitate to higher-degree complex interactions that occur in the in vivo conditions. As the human brain is built of different cell types—neurons, astrocytes, microglia, oligodendrocytes, pericytes, and the epithelial cells, combining them in a culture provides a more useful system for studying complex cell-to-cell interactions that occur in the CNS, in both physiological and pathological conditions.

Co-culturing of cells can be achieved in a 2D cell culture environment, but a more representative approach involves the use of 3D cell culture models that allow establishment of multiple interactions between different cell types and the ECM. Adding multidimensionality to in vitro systems also enables cells to develop distinct phenotypes that are more physiologically relevant. Major advantage of using 3D cultures in in vitro research is the ability to reconstruct 3D organization of cells, and it represents an important step in an effort to imitate normal cell-to-cell and cell-to-ECM interactions. Additional benefit of using 3D in vitro models is the increased viability of difficult-to-culture cells, improved cell-type-specific function and gene expression, and the accumulation of secreted factors in the ECM that could have pathological effects and cannot be studied in 2D cell culture. Cells grown in 3D cultures can self-organize and differentiate, and they allow highly scalable and high-throughput analyses of cell responses and can be used to obtain electrophysiological network activity outputs.

Different types of 3D systems for culturing cells include the scaffold-free, scaffold based, and hybrid culture strategies. Scaffolds are structures that are made of biopolymers organized in a way to imitate the physiological ECM. They are matrices that can be made of hydrogels or can be of solid, porous, and fibrous build. Other than providing the structural support, scaffolds can be enriched with different molecules, e.g., growth factors, thus adding to the similarity of the cultured environment to in vivo conditions.

In both scaffold-based and scaffold-free systems, cells can be cultured in 3D structures. Multicellular aggregates called spheroids are 3D structures that can mimic various as well as tumors [38,39,40]. Neurospheres are neuronal aggregates created from neural progenitor cells that can be manipulated to generate brain-region-specific cell types (e.g., neurons and astrocytes). They have proven to be useful in the neurodevelopment and neurodegenerative diseases research, but one major limitation is the creation of the necrotic core in the central part of the spheres that occurs due to the insufficient perfusion and the lack of vascularization.

First developed in 2008 in the Sasai lab [41], 3D tissue models of cerebral cortex (cerebroids) are becoming more and more used for modeling neurological diseases. As the brain organoid technology is rapidly advancing, recently, it has been also frequently found in the modeling of TBI and stroke. Organoids are developed from ESCs or iPSCs and grown to appropriate dimensions and development stages. They are superior to simple neurospheres, because they have brain-mimetic features and it is possible to reproduce the topological organization of distinct brain regions. Limitations in the cerebroid use are related to the high variability and the lack of reproducibility and the fact that they generally require long-term culturing to achieve sufficient cell growth and maturation. With the improvements in the methodologies and protocols, in due time it will be possible to generate organoids in large enough quantities, with minimal batch-to-batch differences, for them to be used as reproducible models for high-throughput screening research. A limited number of studies on pathological mechanisms and therapeutic interventions in TBI and stroke have thus far used organoids [42,43,44], but the quantities of this kind of research studies are bound to significantly increase in the coming years.

Organs-on-chip technologies, including brain-on-chip technology, also appear very promising [45,46,47,48]. Organs-on-chip are 3D cell culture systems in which miniature tissues are grown inside microfluidic chips that are designed to control the microenvironment of cells. However, there is still a need to increase the reproducibility and standardize these systems.

At this point, there is a general lack of consensus on what would be the optimal methods and culture conditions to generate 3D cultures or brain organoids. Current methods are also expensive and time-consuming, and there is still batch-to-batch variability in organization that may influence the reproducibility of the study results.

### 4.4. Organotypic Slice Cultures

Organotypic slices are used also in the TBI and stroke research as a tool to study the effects of injury on cells that preserve neuronal connections [49]. They not only allow analysis of electric activity in circuits and measurement of calcium changes during injury, but are also amenable to interventions, e.g., by using the optogenetic approach.

In the brain ischemia research, hippocampal organotypic slices are frequently used, and they proved to be useful in studying the pathophysiology of stroke [50,51], neuron-glia interactions [52,53], as well as a useful platform for the screening of the therapeutic interventions, both pharmacological and non-pharmacological [54,55].

However, the technique of obtaining the tissue slices requires that it is cut out, and this trauma itself could be used as an injury model [25]. As an example, it has been found that organotypic slices develop epileptiform activity after a week in culture that resembles changes related to the post-traumatic epilepsy [56]. Additionally, another hindrance in using the slices is in the fact that they are typically dissected from the brains of very young animals with major differences in synaptic physiology and greater synaptic plasticity and that are also more resistant to injurious stimuli [57].

### 4.5. Human Induced Pluripotent Stem Cells

The use of iPSCs technology, specifically human iPSCs, has many advantages in the different-disease/disorder research. This technology was first described in 2007, when the adult human fibroblasts were reprogrammed back to a pluripotent state using specific factors [57]. These cells maintain the genetic features of their parent cells, but with the added property of being able to proliferate easily with the additional possibilities of genetic manipulation.

Even though human iPSCs can be used as a powerful tool to study diseases in many different organs, they are especially useful in neurological disorders research. The reason for this is the fact that it is particularly challenging to obtain human neuronal tissues and cells and also because of the distinctive properties of human CNS.

Some of the advantages in using human iPSCs are the ability to derive specific cell types (neurons, astrocytes, and microglia) from controls and individuals suffering from a certain disorder/disease. Thus, it creates an ideal environment to screen on-target drug effects. They mimic brain development and pathologies better than both human immortalized cancer cell lines and primary rodent cell cultures.

What the use of human iPSCs also provides is the possibility to develop more physiologically relevant and complex assays by establishing both 2D and 3D model systems. At this point, as the iPSCs technology is still in the development stage, it is necessary to refine the protocols in order to optimize and standardize the processes and ensure reliable and reproducible results.

Human iPSCs-derived CNS cells and organoids provide a unique opportunity in the research of both TBI and stroke. Regarding the studies that have thus far utilized this technology, not many have been published. In Table 2, some of the studies using the human iPSCs in the TBI and brain ischemia research are presented.

## 5. Conclusions

In the recent years, a significant shift in the preclinical biomedical research has happened regarding the use of live animals, mainly related to ethical aspects, particularly considering that in vivo experiments usually require the use of a large number of animals. Implementation of experimental strategies that prioritize the use of cells and tissues in the in vitro environment has significantly reduced the number of in vivo studies. In vitro studies certainly have some advantages compared to in vivo experiments, e.g., they allow high-throughput screening of therapeutic approaches, including the use of cells with human-based backgrounds. However, they still cannot completely replicate the complex intricacies of a living organism’s response to disease or injury as well as to therapeutics. This is something that is particularly true in neuroscience research and one of the main reasons why the neuroprotective strategies, which have been proven promising in the preclinical setting, overall failed to show benefits in human studies.

Animal models of TBI and stroke have been used for decades to study the pathophysiological mechanism of these brain disorders as well as to test potential therapeutic approaches. However, even though the preclinically obtained data suggested numerous possible therapies for both TBI and stroke, in the clinical phase studies almost all suggested approaches have failed to produce similar results. This is the reason why an effort needs to be made to improve preclinical testing methods and thus increase the relevancy of results obtained by this type of research studies. This involves both improving the model systems as well as prioritizing the use of cells of human origin, especially with iPSCs as the most promising source of CNS cells for neurotherapeutics discovery.

## Figures and Tables

**Table 1:** Some of the most commonly used immortalized cell lines in in vitro TBI and cerebral ischemia studies.

Cell Line	Origin and Source	Selected References
SH-SY5Y	Human, neuroblastoma cells	[27,28]
NTera (NT2)	Human, neuronally committed teratocarcinoma cell line	[29,30]
PC12	Rat, derived from a pheochromocytoma of the adrenal medulla	[31,32]
C6	Rat, glioma cell line	[33,34]
SVG	Human, immortalized astrocytes	[35]
BV-2	Mouse, murine microglial cell line	[23,36]
N19	Mouse, immortalized oligodendrocytes	[37]

**Table 2:** Summary of the selected studies using in vitro models of brain trauma or ischemia and the human iPSCs.

Model	Origin and Cell Type	References
TBI/stretch injury	Human iPSC-derived neurons	[58]
TBI/stretch injury	Human iPSC-derived neurons	[59]
TBI/blast injury	3D aggregates of human iPSCs (minibrains)	[60]
TBI/compressive injury	Cortical spheroids derived from human iPSCs	[61]
TBI/stretch injury	Human iPSC-derived neurons	[62]
TBI/controlled cortical impact	Human iPSC-derived cerebral organoids	[44,63]
TBI/weight-drop model	Human iPSC-derived neural progenitor cells	[64]
TBI/neurite transection model	Human iPSC-derived neurons	[65]
Oxygen-glucose deprivation/reperfusion	Human brain-derived microvascular endothelial cells from iPSCs	[66]
Hypoxia model	Human iPSC-derived neurons	[67]
Oxygen-glucose deprivation/reperfusion	Human iPSC-derived neurons	[27]
Oxygen-glucose deprivation	Human iPSC-derived neurons in 3D culture	[68]
Hypoxia	Human iPSC-derived neurons	[67]

## Data Availability

The data that support the findings of this study are available upon reasonable request (e.g., research purpose) from the authors.
